# Comparable outcomes in male and female patients undergoing periacetabular osteotomy

**DOI:** 10.1002/jeo2.70761

**Published:** 2026-05-19

**Authors:** Quentin Karisch, Chiara Heller, Justus Stamp, Marco Haertlé, Henning Windhagen, Sufian S. Ahmad

**Affiliations:** ^1^ Department of Orthopedics Hannover Medical School Hannover Germany

**Keywords:** gender differences, outcome, patient‐reported outcome measures, periacetabular osteotomy, sex differences

## Abstract

**Purpose:**

Periacetabular osteotomy (PAO) is an established treatment for DDH with generally favourable outcomes. Although developmental hip dysplasia (DDH) is more prevalant in females, it remains unclear whether male patients achieve comparable patient reported outcome, with some studies suggesting inferior outcome in men. The aim of this study was to evaluate short‐term outcomes of PAO in male versus female hips.

**Methods:**

A prospective PAO registry was utilized and predefined inclusion criteria applied. A total of 282 hips (44 male, 238 female) that had undergone PAO surgery between 2022 and 2024 by a single surgeon were included. Radiographic measures and patient‐reported outcome measures (PROMs), including the University of California, Los Angeles activity scale (UCLA), Hip Disability and Osteoarthritis Outcome Score – Physical Function Shortform (HOOS‐PS), Western Ontario and McMaster Universities Osteoarthritis Index (WOMAC), international hip outcome tool‐12 (iHOT‐12), Harris hip score (HHS), modified Harris Hip Score (mHHS) and Postel‐Merle d'Aubigné (PMA) score, were assessed preoperatively and at final follow‐up and compared between sexes.

**Results:**

Preoperatively, male patients had higher BMI (27.07 vs. 25.01, *p* = 0.019), greater anterior (Anterior wall coverage [AC] 48.09 vs. 41.28, *p* = 0.008) but lower posterior acetabular coverage (Posterior wall coverage [PC] 78.21 vs. 85.77, *p* = 0.010) and more concomitant procedures (54.5% vs. 18.5%, *p* < 0.001). The UCLA was the only PROM showing a significant sex‐related difference in preoperative values. Both sexes improved significantly in most PROMs, with no significant differences in the magnitude of improvement. Likewise, achievement rates of PASS and MCID for mHHS and iHOT‐12 were comparable between sexes. Given the small number of male patients, results should be interpreted with caution due to potential limited statistical power.

**Conclusion:**

The findings of this study demonstrate that there are no significant sex‐related differences in the clinical burden of DDH or in short‐term outcomes after PAO in patients with this diagnosis. Both males and females showed substantial improvement after PAO, indicating that the procedure provides similarly favourable outcomes for both sexes. Based on these results, gender should not influence decision‐making in PAO surgery.

**Level of Evidence:**

Level III.

**Abbreviations**
AC: anterior wall coverage
AI: acetabular index
ANOVA: analysis of variance
BMI: body mass index
DDH: developmental dysplasia of the hip
EI: extrusion index
G‐FJS: German Forgotten Joint Score
HHS: Harris hip score
HOOS‐PS: hip disability and osteoarthritis outcome score – physical function shortform
iHOT‐12: international hip outcome tool – 12
LCEA: lateral centre‐edge angle
MCID: minimal clinically important difference
mHHS: modified Harris hip score
PAO: periacetabular osteotomy
PASS: patient acceptable symptome state
PC: posterior wall coverage
PMA: postel‐Merle d'Aubigné score
PROM(s): patient‐reported outcomes measure(s)
UCLA: University of California, Los Angeles activity score
WOMAC: Western Ontario and McMaster Universities Osteoarthritis Index

## INTRODUCTION

Developmental dysplasia of the hip (DDH) is a disorder of the hip joint that, despite consistent neonatal screening and early treatment, still shows a high prevalence in the adult population [15, 20, 27]. Notably, DDH affects females substantially more frequently than males [7, 13, 17, 31, 44].

A defining characteristic of DDH is insufficient acetabular coverage of the femoral head. Corrective procedures, such as periacetabular osteotomy (PAO), aim to improve functional femoral head coverage by reorienting the acetabulum. Favourable outcomes of the procedure have been demonstrated in numerous studies for a variety of indications over the spectrum of acetabular pathomorphologies [2, 16, 18, 25].

In recent years, patient‐reported outcome measures (PROMs) have gained increasing importance in assessing clinically meaningful outcomes from the patient's perspective [35]. In this context, thresholds such as the patient acceptable symptom state (PASS) and the minimal clinically important difference (MCID) have become important tools for determining the clinical relevance of changes in PROMs [21, 43].

Previous studies have consistently demonstrated improvements in PROMs following PAO for DDH [24]. However, data regarding sex related differences remains limited, and the prevalence of female patients among individuals with DDH may bias existing outcome data [7, 13, 17, 31, 44].

Several studies suggest that male patients may present with distinct preoperative characteristics compared with female patients. These include a higher body mass index (BMI) [10], a higher prevalence of posterior undercoverage [10, 14, 41], more concomitant hip pathologies such as asphericity of the head‐neck junction [10, 45] and milder symptoms compared to females [12].

Few reports have been published addressing sex specific outcomes after PAO. In most studies, relatively small numbers of male patients have been included with heterogeneous results. While some authors report inferior outcomes in male patients [8, 37], others have found no significant sex‐related differences following PAO [3, 28, 29, 30, 32, 38, 39], whereas one study reported higher University of California, Los Angeles activity scale (UCLA) scores in male patients [12].

The aim of the present study was to evaluate the short‐term outcome [1] of PAO in a cohort of symptomatic male hips compared to female hips undergoing PAO.

It was hypothesised that male hips demonstrate poorer outcome scores after PAO, compared to female hips.

## METHODS

**Figure 1:**
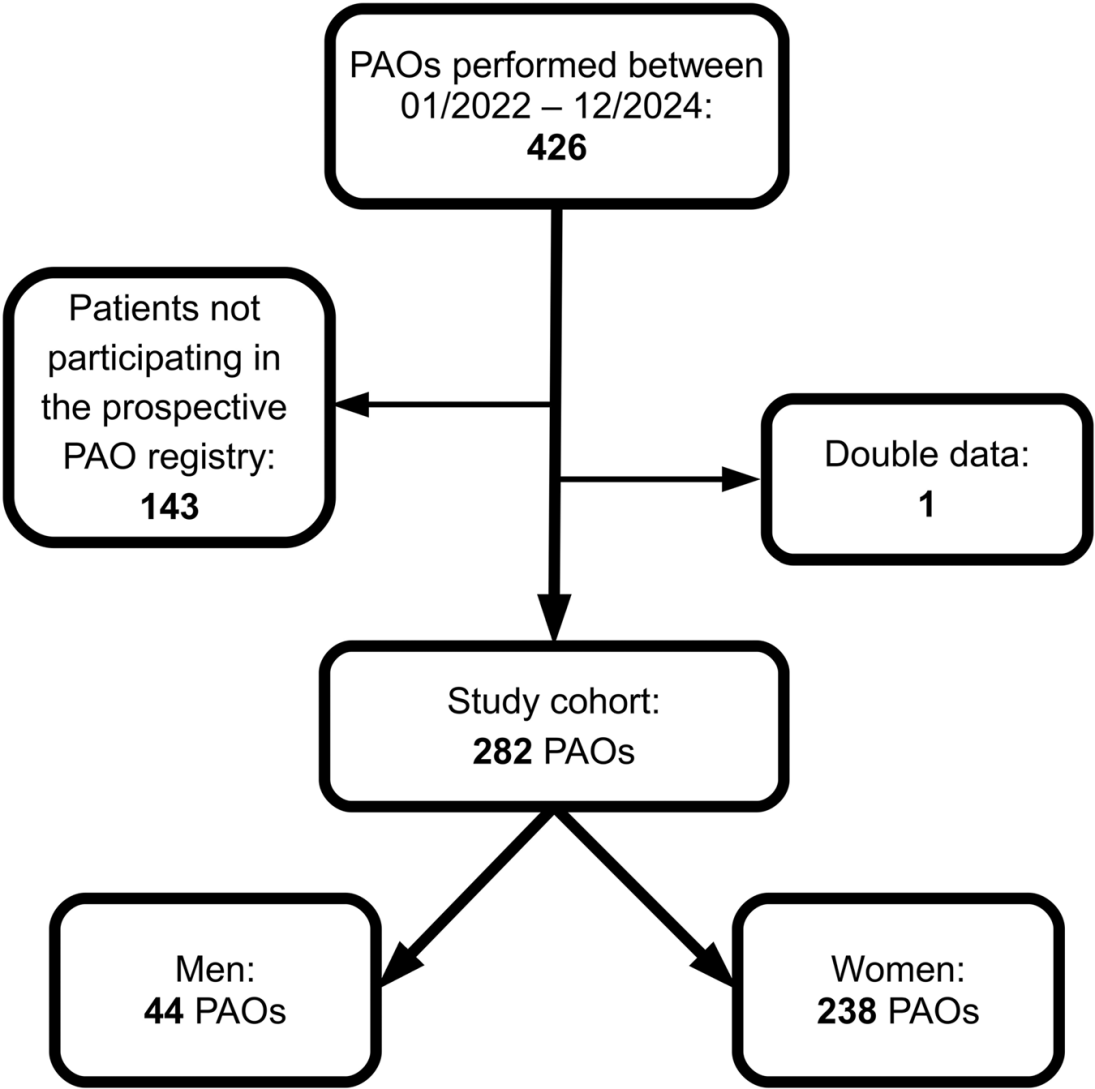
Flowchart demonstrating the selection process of hips included in the final analysis. PAO, periacetabular osteotomy.

A prospective PAO PROM registry was utilised for the conduct of the study. During the study period between January 2022 and December 2024, a total of 426 PAOs were performed by a single surgeon (SSA). Of these, 282 provided consent for the 10‐year follow‐up registry and met the inclusion criteria (Figure 1).

Patients were eligible for inclusion if they underwent PAO for DDH, defined by a lateral centre edge angle of <25°. Patients were excluded if language barriers prevented reliable completion of PROMs.

Participants were stratified according to sex into two groups: male patients with DDH and female patients with DDH.

### Radiographic assessment

All patients underwent standardised preoperative radiographic assessment according to the institutional PAO protocol, including an anteroposterior pelvic radiograph. Hip morphology was evaluated using the following parameters: Lateral centre‐edge angle (LCEA), acetabular index (AI), extrusion index (EI), anterior wall coverage (AC) and oosterior wall coverage (PC). All radiographic measurements were performed by trained orthopaedic surgeons and according to the method described by Tannast et al. [40].

### PROMs and clinical assessment

PROMs were collected using the following validated questionnaires: UCLA, Hip Disability and Osteoarthritis Outcome Score—Physical Function Shortform (HOOS‐PS), Western Ontario and McMaster Universities Osteoarthritis Index (WOMAC), international hip outcome tool‐12 (iHOT‐12), Harris hip score (HHS), modified Harris hip score (mHHS) and postel Merle d'Aubigné (PMA) score. PROM questionnaires were completed independently by the patients. Clinical components of the HHS were assessed and documented by trained medical personnel.

Outcome assessments were obtained preoperatively and at 12, 24 and 36 months postoperatively. The PMA and HHS were assessed at 12 and 24 months postoperatively. For the present analysis, the longest available follow‐up value for each patient was used.

To determine clinically meaningful outcome thresholds, rates of PASS and MCID were calculated for the mHHS and iHOT‐12. The following thresholds were applied: PASS ≥ 71 [42] and MCID ≥ 8 [23] for the mHHS and PASS ≥ 44 [4] and MCID ≥ 13 [26] for the iHOT‐12.

Concomitant procedures performed in addition to PAO were documented.

### Variables and endpoints

The primary endpoint of the study was the PROMs for both male and female hips undergoing PAO at short‐term follow‐up.

### Statistical analysis

Data collection was performed using Microsoft Excel (Microsoft Corp.). Statistical analyses were conducted using IBM Statistics Version 30.0.0.0 (IBM Corp.). Figures were generated using GraphPad Prism Version 11 (GraphPad Software). A prior power analysis was performed using G*Power (version 3.1.9.7). Based on an assumed moderate power of 80%, at least 64 patients per group were required to detect a statistically significant between‐group difference.

This study was not specifically powered to detect small sex‐related differences in postoperative outcomes following PAO. Given the available sample size, the analysis may be underpowered to identify subtle between‐group differences in postoperative outcomes. Therefore, the absence of statistically significant differences should be interpreted cautiously and does not imply equivalence between sexes.

Continuous variables were tested for normality using the Shapiro–Wilk test and are presented as mean ± standard deviation. Homogeneity of variances was assessed using Levene's test. Categorical variables are shown as counts and percentages. For group comparisons, the unpaired *t*‐test was used for normally distributed data when variances were equal. Otherwise, Welch's *t*‐test was applied. The Mann–Whitney *U*‐test was used for nonnormally distributed continuous variables. Fisher's exact test was used for categorical variables when expected cell counts were small. For paired comparisons, the paired *t*‐test for normally distributed data and the Wilcoxon signed‐rank test for nonnormally distributed data. Effect sizes were calculated for all primary comparisons. A binary logistic regression analysis was performed to evaluate the association between AC, PC and simultaneous interventions. Sex was included as a covariate to adjust for potential confounding. To address potential demographic heterogeneity between male and female patients, an analysis of covariance (ANCOVA) was performed for each PROM at final follow‐up. Postoperative scores were defined as dependent variables, sex as the fixed factor and the respective preoperative PROMs as covariates. Additional covariates included age, BMI, side, concomitant procedures and radiographic parameters. The assumption of homogeneity of regression slopes was tested by including an interaction term between sex and the respective preoperative PROM. As this was not significant, interaction terms were removed from the final models. A *p* < 0.05 was considered statistically significant.

### Ethical statement

This study was conducted in accordance with the principles of the Declaration of Helsinki. Ethical approval was obtained from the local ethics committee of Hannover Medical School (10450_BO_K_2022).

The study was conducted at the Department of Orthopedics, Hannover Medical School, Anna‐von‐Borries‐Strasse 1‐7, 30625 Hannover, Germany.

## RESULTS

### Demographic data

Male patients demonstrated a significantly higher BMI compared with female patients (BMI_Male _= 27.07, BMI_Female_ = 25.01, *p* = 0.019). In addition, males showed significantly greater anterior acetabular coverage (AC_Male _= 48.09%, AC_Female_ = 41.28%, *p* = 0.008) and reduced posterior coverage (PC_Male _= 78.21%, PC_Female_ = 85.77%, *p* = 0.010). Concomitant procedures performed during PAO were significantly more common in male patients than in female patients (54.5% vs. 18.5%, *p* < 0.001) (Table 1). These additional procedures included offset corrections (47.7% vs. 13.4%), contralateral implant removals (4.5% vs. 1.3%), torsional corrections (0% vs. 1.7%), combined offset and torsional corrections (2.3% vs. 0.4%), contralateral joint infiltrations (0% vs. 0.8%), exploratory arthrotomies (0% vs. 0.4%) and intertrochanteric osteotomies (0% vs. 0.4%). In the binary logistic regression, AC was significantly associated with simultaneous interventions (OR = 19.59, 95% CI 2.80–136.87, *p* = 0.003), whereas PC showed no significant association (*p* = 0.382). Sex was independently associated with simultaneous interventions (OR = 5.14, 95% CI 2.51–10.55, *p* < 0.001). The model correctly classified 79.4% of cases.

**Table 1:** Descriptive data: Dysplasia in male vs female patients.

	Male		Female		*p*‐value	Effect size
	*N*	Mean ± SD	*N*	Mean ± SD		
Number of patients						
	44/282 (16%)		238/282 (84%)			
Average age (years)						
	44	29.98 ± 6.67	238	29.05 ± 7.93	0.347**	0.056
Body mass index (kg/m^2^)						
	42	27.07 ± 5.12	223	25.01 ± 4.64	0.019**	0.144
Side						
Right	22/44 (50%)		117/238 (49%)		1.000*	0.006
Left	22/44 (50%)		121/238 (51%)			
Additional intervention during PAO						
Yes	24/44 (54.5%)		44/238 (18.5%)		<0.001*	0.306
No	20/44 (45.5%)		194/238 (81.5%)			
Preoperative radiographic measurements						
LCEA (°)	44	20.65 ± 8.65	237	18.30 ± 7.25	0.156**	0.085
AI (°)	44	14.21 ± 6.48	237	13.72 ± 6.91	0.479**	0.042
EI (%)	44	27.30 ± 7.39	237	27.19 ± 7.28	0.553**	0.035
AC (%)	44	48.09 ± 15.51	237	41.28 ± 15.61	0.008**	0.158
PC (%)	44	78.21 ± 15.94	237	85.77 ± 18.14	0.010***	0.425

*Note*: Effect sizes: *φ* for Fisher's exact test; Cohen's *d* for unpaired *t*‐test; for Mann–Whitney *U*‐test (CI(Cohend's *d*)_PC_: [0.101; 0.748]). Abbreviations: AC, anterior wall coverage; AI, acetabular index; CI, confidence interval; EI, extrusion index; LCEA, lateral centre‐edge angle; PC, posterior wall coverage; SD, standard deviation.

* Fisher's exact test;

** Mann–Whitney‐*U*‐test;

*** Unpaired *t*‐test.

### Within‐group and between‐group outcome comparison

**Figure 2:**
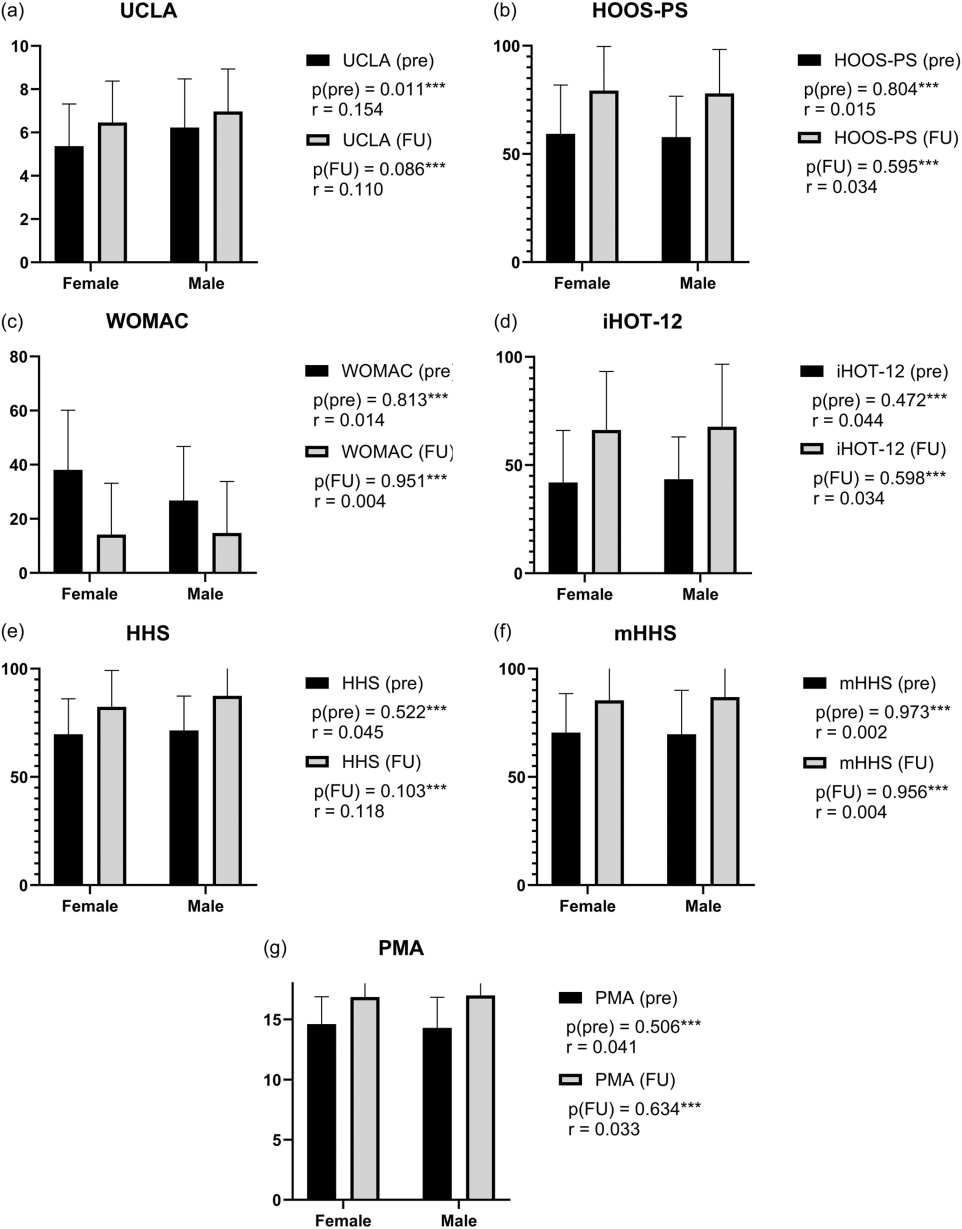
(a) Comparison of pre and FU on the UCLA between male patients with dysplasia (mean_pre_ = 6.28, SD_pre_ = 2.25, mean_FU_ = 6.97, SD_FU_ = 1.97, *p* = 0.095**) and female patients with dysplasia (mean_pre_ = 5.37, SD_pre_ = 1.95, mean_FU_ = 6.46, SD_FU_ = 1.92, *p* < 0.001**). (b) Comparison of pre and FU scores on the HOOS‐PS between male patients with dysplasia (mean_pre_ = 57.79, SD_pre_ = 18.91, mean_FU_ = 77.97, SD_FU_ = 20.33, *p* < 0.001*, *r* = −1.348, CI(MD): [–23.45; –14.05]) and female patients with dysplasia (mean_pre_ = 59.28, SD_pre_ = 22.56, mean_FU_ = 79.30, SD_FU_ = 20.39, *p* < 0.001*, *r* = –0.836, CI(MD): [–21.50; –15.32]). (c) Comparison of pre and FU scores on the WOMAC between male patients with dysplasia (mean_pre_ = 26.75, SD_pre_ = 20.04, mean_FU_ = 14.75, SD_FU_ = 19.05, *p* < 0.001*, *r* = 0.633, CI(MD): [4.11; 15.82]) and female patients with dysplasia (mean_pre_ = 28.11, SD_pre_ = 22.05, mean_FU_ = 14.19, SD_FU_ = 18.95, *p* < 0.001**, *r* = 0.589). (d) Comparison of pre and FU scores on the iHOT‐12 between male patients with dysplasia (mean_pre_ = 43.47, SD_pre_ = 19.55, mean_FU_ = 67.70, SD_FU_ = 28.94, *p* < 0.001*, *r* = –1.102, CI(MD): [–30.74; –16.30]) and female patients with dysplasia (mean_pre_ = 41.98, SD_pre_ = 24.03, mean_FU_ = 66.19, SD_FU_ = 27.12, *p* < 0.001*, *r* = –0.792, CI(MD): [–26.02; –18.16]). (e) Comparison of pre and FU scores on the HHS between male patients with dysplasia (mean_pre_ = 71.47, SD_pre_ = 15.87, mean_FU_ = 87.46, SD_FU_ = 14.88, *p* = 0.003*, *r* = –1.024, CI(MD): [–18.30; –7.04]) and female patients with dysplasia (mean_pre_ = 69.72, SD_pre_ = 16.37, mean_FU_ = 82.36, SD_FU_ = 16.83, *p* < 0.001*, *r* = –0.832, CI(MD): [–17.36; –11.10]). (f) Comparison of pre and FU scores on the mHHS between male patients with dysplasia (mean_pre_ = 69.68, SD_pre_ = 20.37, mean_FU_ = 86.87, SD_FU_ = 14.15, *p* < 0.001**, *r* = 0.713) and female patients with dysplasia (mean_pre_ = 70.45, SD_pre_ = 18.09, mean_FU_ = 85.35, SD_FU_ = 16.07, *p* < 0.001**, *r* = 0.641). (g) Comparison of pre and FU scores on the PMA between male patients with dysplasia (mean_pre_ = 14.30, SD_pre_ = 2.55, mean_FU_ = 17.00, SD_FU_ = 1.54, *p* < 0.001**, *r* = 0.834) and female patients with dysplasia (mean_pre_ = 14.61, SD_pre_ = 2.28, mean_FU_ = 16.86, SD_FU_ = 1.63, *p* < 0.001**, *r* = 0.665). *paired *t*‐test; **Wilcoxon‐test; *** Mann‐Whitney‐*U*‐test. Effect sizes (*r*): paired Cohen's *d* for paired *t*‐test; *r* $=Z/\sqrt{N_{\text{total}}}$ for Mann–Whitney U; $r\;=Z/\sqrt{N_{\text{pairs}}}$ for Wilcoxon signed‐rank test. CI, confidence interval; FU, follow‐up; HHS, Harris hip score; HOOS‐PS, hip disability and osteoarthritis outcome score—physical function shortform; iHOT‐12, international hip outcome tool—12; MD, mean differences; mHHS, modified Harris hip score; PMA, postel Merle d'Aubigné score); Pre, preoperative; *r*, effect size; SD, standard deviation; UCLA, University of California and Los Angeles activity scale; WOMAC, Western Ontario and McMaster Universities Osteoarthritis Index.

At baseline, only the UCLA differed significantly between groups, with male patients demonstrating higher preoperative scores (*p* = 0.011) (Figure 2).

Follow‐up rates (male/female) were as follows: UCLA (84%/87%), HOOS‐PS (84%/87%), WOMAC (83%/85%), iHOT‐12 (84%/86%), HHS (66%/69%), mHHS (82%/87%) and PMA (64%/76%).

The mean follow‐up duration in months (male/female) was: UCLA (21.08/19.17), HOOS‐PS (21.08/19.22), WOMAC (20.76/19.29), iHOT‐12 (21.08/19.22), HHS (19.29/16.93), mHHS (21.00/19.22) and PMA (19.29/17.00).

Both male and female patients demonstrated significant improvements in nearly all PROMs at follow‐up compared with their preoperative values. However, in male patients, the UCLA did not show statistically significant improvement (*p*_UCLA _= 0.095).

When comparing the magnitude of improvement, defined as the difference between follow‐up and baseline values, no statistically significant sex‐related differences were observed across the evaluated PROMs (Table 2).

**Table 2:** Differences between the PROMs preoperatively versus follow‐up at dysplasia in male vs female patients.

	Male		Female		*p*‐value	Effect size
	*N*	Δ Preop versus FU (Mean ± SD)	*N*	Δ Preop versus FU (Mean ± SD)		
UCLA						
	36	0.50 ± 1.66	198	0.95 ± 1.76	0.211**	0.082
HOOS‐PS						
	36	18.75 ± 13.91	198	18.41 ± 22.03	0.929***	−0.016
WOMAC						
	35	−10.58 ± 15.24	190	−12.11 ± 19.13	0.721**	0.024
iHOT‐12						
	36	23.52 ± 21.33	196	22.09 ± 27.90	0.771***	−0.053
HHS						
	21	12.67 ± 12.37	117	14.23 ± 17.11	0.620****	0.126
mHHS						
	36	16.27 ± 20.68	200	12.84 ± 17.40	0.641**	0.030
PMA						
	28	2.82 ± 2.02	167	2.01 ± 2.45	0.065**	0.132

*Note*: Effect sizes: Cohen's d for unpaired and Welch t‐test; r = Z/Ntotal for Mann‐Whitney U(CI(Cohen's d)_HOOS‐PS_: [−0.371; 0.339]; CI(Cohen's d)_iHOT‐12_: [−0.380; 0.331]; CI(Cohen's d)_HHS_ [‐0.341; 0.591]). Abbreviations: CI, confidence interval; FU, follow‐up; HHS, Harris hip score; HOOS‐PS, hip disability and osteoarthritis outcome score—physical function shortform; iHOT‐12, International hip outcome tool—12; mHHS, modified Harris hip score; PMA, postel Merle d'Aubigné score; PROM, patient‐reported outcomes measure; SD, standard deviation; UCLA, University of California and Los Angeles activity scale; WOMAC, Western Ontario and McMaster Universities Osteoarthritis‐Index; Δ, difference.

** Mann–Whitney‐*U*‐test;

*** unpaired *t*‐test;

**** Welch‐*t*‐test.

In unadjusted analyses, slight differences in postoperative PROMs between male and female patients were observed. However, after adjustment for baseline PROMs and relevant demographic and radiographic covariates using ANCOVA, no significant differences between sexes were found for any outcome measure at final follow‐up (UCLA: *p* = 0.563; HOOS‐PS: *p* = 0.330; WOMAC: *p* = 0.662; iHOT‐12: *p* = 0.542; HHS: *p* = 0.454; mHHS: *p* = 0.220; PMA: *p* = 0.111). Across all models, the respective preoperative PROM was a significant predictor of postoperative outcome (all *p* < 0.001), whereas the influence of other covariates was small and inconsistent. A significant interaction between sex and preoperative score was observed for HOOS‐PS (*p* = 0.017; partial *η*² = 0.027) and iHOT‐12 (*p* = 0.009; partial *η*² = 0.033).

Similarly, the proportions of patients achieving PASS and MCID for the mHHS and iHOT‐12 did not differ statistically significant between male and female patients (Table 3).

**Table 3:** Rate of PASS and MCID for mHHS and iHOT‐12: Dysplasia in male versus female patients.

	mHHS			iHOT‐12			
	Male	Female	*p*‐value (Effect size)	Male	Female		*p*‐value (Effect size)
PASS rate	PASS mHHS = 71				PASS iHOT‐12 = 44		
Pre	24/44 (55%)	123/230 (54%)	1.000* (0.008)	19/43 (44%)	96/227 (42%)		0.867* (0.014)
Follow‐up	33/36 (92%)	174/206 (85%)	0.315* (0.073)	27/37 (73%)	157/206 (76%)		0.680* (0.027)
MCID rate	MCID mHHS = 8				MCID iHOT‐12 = 13		
	23/36 (64%)	122/200 (61%)	0.853* (0.021)	25/36 (69%)	125/196 (64%)		0.573* (0.043)

*Note*: Effect sizes: *φ* for Fisher's exact test. Abbreviations: iHOT‐12, international hip outcome tool—12; MCID, minimal clinically important difference; mHHS, modified Harris hip score; PASS, patient acceptable symptom state; pre, preoperative.

* Fisher's exact test.

## DISCUSSION

The present study provides consistent, methodologically well‐controlled evidence underlining that patient sex is not a major determinant of PROMs following PAO in a large, well‐characterised cohort. Both male and female patients demonstrated comparable improvements across most PROMs, with no evidence of clinically meaningful sex‐related differences in the magnitude of improvement after adjustment for relevant covariates.

A significant interaction between sex and preoperative baseline scores was observed for HOOS‐PS and iHOT‐12, indicating that baseline status may differentially influence postoperative PROMs in male and female patients. However, effect sizes were small, suggesting limited clinical relevance. Overall, sex did not meaningfully affect adjusted postoperative outcomes when accounting for baseline scores and relevant covariates.

Male patients demonstrated higher preoperative UCLA scores compared with female patients. This may be related to the activity profile captured by the UCLA score, which primarily reflects sports participation. Differences in baseline activity levels between sexes may therefore explain this finding [33, 34].

The ANCHOR‐Group reported that male sex was predictive of lesser improvement after PAO [8]. In contrast, the present study did not demonstrate a clinically meaningful association between sex and postoperative PROMs after adjustment for baseline values and relevant covariates. Several factors may account for this discrepancy. First, the ANCHOR study represents a large multicenter cohort with multiple surgeons and institutions, whereas this study reflects a single‐surgeon series within a highly standardised clinical setting. This likely reduces variability in surgical technique and perioperative management, which may otherwise confound the relationship between sex and outcome. Second, although both cohorts included concomitant procedures to address intraarticular and femoral‐sided pathology, the extent and indication for these interventions may differ substantially between a multicenter registry and a single‐surgeon series. In the present study, male patients more frequently underwent additional procedures, suggesting a more consistent surgical management of complex deformities, which may attenuate previously reported sex‐related differences. Third, differences in statistical methodology max contribute to the divergent finding. While sex emerged as an independent predictor in the multivariate regression models of the ANCHOR study, our analyses indicate that baseline PROMs are the primary determinants of postoperative outcome, with only minor and inconsistent contributions from sex. Taken together, these findings suggest that the influence of sex on PAO outcomes may be context‐dependent and influenced by surgical consistency, cohort heterogeneity and analytical approach.

In contrast to the findings of Fischer et al., except for the UCLA no significant preoperative sex‐related differences were observed in any PROMs [12].

Overall, our findings are consistent with several previous studies reporting that patient sex does not significantly influence clinical outcomes after PAO [3, 12, 28, 29, 30, 32, 36, 38, 39]. Compared with previous literature, the present study adds value through comprehensive PROM assessment and adjusted analyses. In addition, all procedures were performed by a single surgeon within a short 2‐year period in a standardised healthcare setting in Germany, which enhances internal validity by reducing variability in surgical and perioperative factors.

The absence of statistically significant sex differences should be interpreted with caution, as the relatively small number of male patients may have limited the power to detect between‐group differences. This imbalance reflects the known sex distribution of hip dysplasia, which predominantly affects female patients [7, 13, 17, 31, 44] and is consistent with prior studies reporting similarly small or even smaller male subgroups [3, 8, 10, 12]. Therefore, the lack of significant differences should not be interpreted as evidence of equivalence.

With regard to preoperative radiographic parameters of hip morphology, significant sex‐related differences were observed in anterior and posterior acetabular coverage. Female patients demonstrated increased acetabular anteversion, whereas male patients more frequently exhibited deficiencies in posterior coverage. These findings are consistent with previous studies describing sex‐specific differences in hip morphology [10, 14, 41].

In addition to acetabular coverage, BMI differed significantly between male and female patients in the present cohort, as previously shown by other authors [10]. Although BMI has been reported to influence preoperative PROMs [22], this difference did not translate into sex‐specific differences in clinical outcomes. One possible explanation is that the observed BMI difference of approximately two units may be too small to produce clinically meaningful effects. This finding is in line with the notion that patient sex itself does not appear to substantially influence PROMs after PAO. Future studies specifically investigating the relationship between BMI and postoperative outcomes may therefore be of interest.

Finally, additional procedures performed during PAO were significantly more common in male patients in the present study. This observation is consistent with previous reports describing a higher prevalence of concomitant femoral pathologies in male patients with DDH [10, 45]. In the present analysis, sex remained an independent predictor of additional procedures even after adjusting for AC and PC.

The rates of patients achieving PASS for the mHHS and iHOT‐12 were comparable to or higher than those reported in the existing literature, further supporting the effectiveness of PAO for the treatment of DDH in both male and female patients [26, 42].

## LIMITATIONS

Several limitations of the present study should be considered when interpreting the results.

First, the follow‐up data represent short‐term outcome results.

Second, although the overall cohort comprised 282 PAOs, the number of male patients was comparatively small. A total of 44 male hips were included preoperatively, and approximately 37 male hips were available at follow‐up for most PROMs. Based on the a priori power calculation, a minimum of 64 participants per group would be required to achieve a statistical power of 80%, suggesting that the study may be underpowered to detect small sex‐related differences.

Third, despite significant baseline differences between sexes in acetabular morphology, BMI and concomitant procedures, no sex‐related differences in postoperative outcomes were observed after adjustment for relevant covariates.

Follow‐up rates were high for most PROMs (80%–90%), but lower for the HHS and PMA (50%–70%). This was mainly due to missing clinical examinations required for score calculation. For the PMA, differences in follow‐up rates between sexes were driven by the smaller male sample sizes, where few missing cases had a relatively larger impact on percentages.

Finally, several classical hip PROMs were originally developed for older patient populations [5, 6, 9, 11, 19], whereas the present cohort had a mean age of approximately 30 years. The UCLA, which assesses physical activity and may be more relevant in younger patients, showed statistically significant baseline sex differences.

## CONCLUSION

In conclusion, the findings of this study demonstrate that there are no significant sex‐related differences in the clinical burden of DDH or in short‐term outcomes following PAO. Both male and female patients demonstrated substantial improvements in PROMs after PAO, indicating that the procedure provides similarly favourable outcomes for both sexes.

## AUTHOR CONTRIBUTIONS

**Quentin Karisch**: Study design; statistical analysis; manuscript writing and revision. **Sufian S. Ahmad**: Study design; statistical analysis; manuscript writing and revision. **Justus Stamp**: Graphics; manuscript writing and revision. **Chiara Heller**: Graphics; manuscript writing and revision. **Marco Haertlé**: Graphics; manuscript writing and revision. **Henning Windhagen**: Graphics; manuscript writing and revision.

## CONFLICT OF INTEREST STATEMENT

The authors declare no conflicts of interest.

## ETHICS STATEMENT

Ethical approval was obtained from the local ethics committee of Hannover Medical School (10450_BO_K_2022). Written informed consent was obtained.

## Supporting information

Supporting File

## Data Availability

Raw data is available upon request.
