# Supplementary material for: Comparable outcomes in male and female patients undergoing periacetabular osteotomy
Source: J Exp Orthop. 2026 May 19;13(2):e70761. doi: 10.1002/jeo2.70761 (PMC13184828; doi:10.1002/jeo2.70761)
Supplement: Supplementary file 1 — Supporting File [file JEO2-13-e70761-s001.docx]

Distribution of PROMs across different data collection time points

|  | | | | | |
| --- | --- | --- | --- | --- | --- |
| **PROM** | **Sex** | **Total (n)** | **12 Mo (n, %)** | **24 Mo (n, %)** | **36 Mo (n, %)** |
|  |  |  |  |  |  |
|  |  |  |  |  |  |
| ***UCLA*** | Male | 37 | 14 (38%) | 18 (49%) | 5 (13%) |
|  | Female | 206 | 102 (50%) | 85 (41%) | 19 (9%) |
|  |  |  |  |  |  |
| ***HOOS-PS*** | Male | 37 | 14 (38%) | 18 (49%) | 5 (13%) |
|  | Female | 206 | 101 (49%) | 86 (42%) | 19 (9%) |
|  |  |  |  |  |  |
| ***WOMAC*** | Male | 37 | 15 (41%) | 17 (46%) | 5 (13%) |
|  | Female | 204 | 99 (49%) | 86 (42%) | 19 (9%) |
|  |  |  |  |  |  |
| ***iHOT-12*** | Male | 37 | 14 (38%) | 18 (49%) | 5 (13%) |
|  | Female | 206 | 101 (49%) | 86 (42%) | 19 (9%) |
|  |  |  |  |  |  |
| ***HHS*** | Male | 28 | 11 (39%) | 17 (61%) | 0 (0%) |
|  | Female | 163 | 96 (59%) | 67 (41%) | 0 (0%) |
|  |  |  |  |  |  |
| ***mHHS*** | Male | 36 | 14 (39%) | 17 (47%) | 5 (14%) |
|  | Female | 206 | 101 (49%) | 86 (42%) | 19 (9%) |
|  |  |  |  |  |  |
| ***PMA*** | Male | 28 | 11 (39%) | 17 (61%) | 0 (0%) |
|  | Female | 180 | 105 (58%) | 75 (42%) | 0 (0%) |

Abbreviations: PROM=Patient-reported outcome measure; UCLA=University of California and Los Angeles activity scale; HOOS-PS=Hip Disability and Osteoarthritis Outcome Score – Physical Function Shortform; WOMAC=Western Ontario and McMaster Universities Osteoarthritis-Index; iHOT-12=International hip outcome tool – 12; HHS=Harris hip score; mHHS=Modified Harris hip score; PMA=Postel Merle d’Aubigné score
